# Comparison of anti-Mullerian hormone level between uterine artery embolization and myomectomy in uterine fibroma

**Published:** 2015

**Authors:** F Keshavarzi, M Salehi, A Mansouri

**Affiliations:** * Department of Radiology, Imam Reza Hospital, Kermanshah University of Medical Sciences, Kermanshah, Iran,; ** Department of Gynecology and Obstetrics, Imam Reza Hospital, Kermanshah University of Medical Sciences, Kermanshah, Iran,; *** Kermanshah University of Medical Sciences, Kermanshah, Iran

**Keywords:** Anti-Mullerian hormone, embolization of uterine artery, myomectomy, uterine fibroma

## Abstract

**Background:** Uterine fibroma was a general gynecologic condition. When pharmacological therapies fail, surgical interferences such as myomectomy, hysterectomy, or embolization of uterine artery (UAE) are used as that state. This study aimed to compare hormone of anti-Mullerian measure among two methods of UAE, myomectomy into the treatment of uterine fibroma.

**Material and Method:** In this clinical trial held in Imam Reza Hospital of Kermanshah, 40 cases by uterine fibroma were entered into the group of UAE (20 cases) and myomectomy (20 cases). Anti-Mullerian hormone levels were measured twice (ago and later therapeutic interventions) using the Monobinal kit. The information are examined by the SPSS (ver. 20.0) software by applying the Leven’s test, paired and independent t-test, Wilcoxon, and Mann-Whitney tests.

**Results:** There is n't matter variation in terms of age between two groups (P> 0.05). Not important variation is recognized regarding anti-Mullerian hormone level before and after six months the medical intervention in either group (P> 0.05). Also, no important variation was detected among the 2 groups in terms of the anti-Mullerian hormone level (P= 0.58).

**Conclusion:** The results obtained demonstrated that where is not statistically important variation among UAE and myomectomy by in terms of anti-Mullerian hormone, which reflects ovarian capacity. Therefore, UAE, which is a less invasive method, can be a suitable substitute for surgical methods in the therapy of significant uterine fibroids between women of the sexual period.

## Introduction

Uterine fibroid (fibroma) is a non-cancerous tissue growth with elastic property in the uterine wall. It is example from the very general gynecologic disorders. Significant fibroids of uterine were associated with considerable complications and affect 20-40% of childbearing age women [**1**]. The most common symptom, which necessitates treatment of fibroids, comprises menorrhagia, which can potentially lead to iron deficiency anemia [**2**].

When symptoms of fibroids progress and medical treatments fail, it may become necessary to implement the intervention. Considering the location of fibroids, surgical methods such as myomectomy or hysterectomy were performed in the past. However, in recent years less invasive methods like embolization of uterine artery (UAE) has been applied. In the past decade, UAE has been utilized as an option to medical methods to reduce the irregular bleeding of uterine. As the best non-surgical method, UAE was first introduced by Ravina in 1995 [**3**].

Many randomized clinical tests have shown that at 24-months follow-up, UAE was associated with comparable outcome with hysterectomy and myomectomy in treatment of uterine fibroids [**4**]. However, ovarian failure, as a complication of UAE, has created some concern about UAE [**4**]. Therefore, studies have targeted this complication of UAE.

The real rate of ovarian failure after UAE is not known. But, in some studies it has been reported at less than two percent [**5**]. One of the diagnostic methods for persistent ovarian failure is raised serum LH and FSH levels [**6**]. Mara et al. (2008) showed that the FSH level was significantly higher in those treated by UAE when compared to myomectomy group [**7**]. In contrast, some studies advocated that UAE did not result in premature ovarian failure [**8**]. For instance, the reports made by Rashid et al. (2010) also Hovsepian et al. (2006) did not mention a important variation in terms of at follow-up period after UAE and surgical intervention [**8**,**9**].

Another diagnostic method to diagnose ovarian failure is the measurement of anti-Mullerian hormone (AMH) level [**7**]. This hormone has a significant role within this ovarian function after birth and was included within primary follicle development; in fact, AMH level is associated via the amount of ovarian follicles [**7**]. This hormone reaches its peak level after puberty and gradually decreases during ovulation periods [**10**]. Diminished ovarian reserve could be determined better and more precisely with the measurement of AMH. AMH has been identified as a reliable marker for ovarian reserve, in particular antral follicles [**11**]. In opposition on LH and FSH, it is no needed to do an AMH measurement on the third day of the menstrual period. In limited former studies, most studied subjects were at pre-menopausal age and the possibility of early ovarian failure was emphasized. In much limited studies, the ovarian capacity in younger patients has been assessed. In addition, ovarian the potential is defined with scaling LH and FSH, not AMH. Since the studies outcomes about ovarian capacity after myomectomy and UAE is controversial and there is limited knowledge about that analogy of ovarian capacity among myomectomy and UAE with 6-months follow-up, this study is conducted for the purpose of analyzing AMH among myomectomy and UAE in the uterine fibroma therapy.

## Material and Method

This was a clinical trial, which, after being verified by the Ethics Committee of Medical Sciences Kermanshah University, is done in the Gynecology Department of Imam Reza Hospital. Using a assurance of 99% and power of 95% and mean (±SD) of AMH in myomectomy and UAE groups of 8.9 (±0.7) and 9.9 (±0.1), the least example measurement is calculated as having 20 persons in each group. 

Measures were undertaken to keep the names of patients confidentially and to avoid any cost on them. Also, in the origin of the research, the details of the interventions were defined with the gynecologist and resident to the patients. Then the patients voluntarily decided to receive UAE or myomectomy, while considering the required criteria for receiving the treatments. In addition, the patients were categorized in four age groups including 20-25, 25-30, 30-35, and 35-40 years and were matched in the 2 group. Demographic and background information (age, hospitalization duration, pain severity after one week, re-intervention, the cost, and time elapsed for the sick for return to work) also clinical and laboratory findings were gathered by the resident and documented in a checklist. AMH measurement was done by the Monobinal kit and all laboratory tests were done at Razi Laboratory. AMH level was measured before the interventions. Pain severity was determined at third post-intervention day. The hospitalization period is texted. The phone number of the resident was delivered to all patients to contact her in case of facing any problem after discharge from hospital. 

Six months later, the patients were contacted by the resident and were asked to present into the clinic for a free consultation and physical examination. At that visit, the AMH level also serum hemoglobin levels were measured. Also, the patients were asked about pain, additional treatments such as hormonal agents, re-intervention, re-presentation to hospital, and time period required to return to work.

The gathered data were entered in SPSS (ver. 20.0) software. The descriptive indices including average and regular variation are utilized to report the results. The examinations are accomplished by employing the Leven’s test, paired and independent t-test, Wilcoxon, and Mann-Whitney U test. The importance level was rated at 0.05.

## Results

The sample studied included 40 women with the age range of 27-40 years. They are split into 2 group: UAE (20 cases) and myomectomy (20 cases). As presented in **Tab. 1**, the average age of the myomectomy group was 35.5 and in UAE group this was 34.55, by not important variation (P= 0.968). Pain score in myomectomy group was 6.3 and in UAE, it was 7.2. Hospitalization duration is notably larger in the group of myomectomy (P< 0.001). However, the cost of UAE was higher than the myomectomy (**Table 1**). 

**Table 1:** Mean (SD) of background variables in uterine artery embolization (UAE) and myomectomy groups

		Myomectomy	UAE	P value
Age	Mean (SD)	35.50 (3.88)	34.55 (3.94)	0.968
Pain	Mean (SD)	6.35 (2.03)	7.20 (1.70)	0.160
Hospitalization (day)	Mean (SD)	3.25 (0.444)	2.05 (0.394)	< 0.001
Treatment costs (Rials)	Mean (minimum-maximum)	297,500 (180,000-500,000)	270,000 (500,000-5,000,000)	< 0.001
Time is needed to turn to post	Mean (minimum-maximum)	10 (5-20)	8.05 (2-12)	0.289

Regarding requirement for re-intervention, follow-up at six months showed that no patient required re-intervention.

The Wilcoxon test results about the variety in AMH level before and six months after the interventions explained not important variety in either groups (P= 0.07). Also, regarding the the Kolmogorov–Smirnov test, AMH level before and after six months had a normal distribution and no statistically important variation was seen (P= 0.839). 

Also, the analyses showed that no significant difference existed between UAE and myomectomy groups regarding AMH level (**Table 2**). 

**Table 2:** Anti-Mullerian hormone (AMH) level before and after six months in uterine artery embolization (UAE) and myomectomy groups

Treatment group	AMH level	Minimum	Maximum	Mean	Standard deviation	P value	P value
Myomectomy (N= 20)	Before intervention	0.12	2.20	2.91	4.42	0.070	0.58
	After intervention	0.1	21	3.05	4.62		
UAE (N= 20)	Before intervention	0.1	7.11	2.24	2.97	0.839	
	After intervention	0.1	20.8	2.14	2.14		

## Discussion

This study was done by the purpose of comparing AMH level among myomectomy and UAE groups in treatment of uterine fibroma. Myomectomy is a standard surgical option for female with fibroma of symptomatic uterine who desire to maintain productivity and do not respond to medical treatment. UAE was a less invasive method in the therapy of the disease. Since there is limited evidence about the ovarian capacity at 6-months follow-up after myomectomy and UAE, the research was done at Imam Reza Hospital of Kermanshah.

In this clinical trial, the 2group did not have an important variation in terms of age. The 2 groups are balanced regarding years; that is an advantage of this study. This variable has not been estimated in other research.

Not important variation is seen regarding pain score among the 2 group, though patients in UAE experienced more severe pain. Hehenkamp et al. (2006) and Edwards et al. (2007) stated that pain in that beginning 24-hours later the mediation was significantly less severe in UAE then in the group of hysterectomy [**12**,**13**], which was not in compliance by our results. However, generally speaking, pain severity is similar among the 2 groups. According to Mara et al. (2008), pain and nausea were not mentioned as main problems later the mediation and these two issues were comparable in surgery groups and UAE, but bloodshed, ache, and pelvic pressure were more common in the group of UAE than the group of hysterectomy [**7**]. In Volker et al. (2007) study, severe hemorrhage was more prominent in UAE than in hysterectomy, but not important variety was observed regarding other complication such as pain and pelvic pressure [**14**]. 

In terms of hospitalization, a significant difference was seen between the two studied groups. This time was shorter in UAE than in the myomectomy group. Likewise, the hospitalization period was shorter in UAE compared to myomectomy and hysterectomy groups according to Hehenkamp et al. (2006), Edwards et al. (2007), Pinto et al. (2003) studies [**4**,**13**,**15**]. In Razavi et al. study (2003), the hospitalization time in UAE was shorter [**16**].

In this running research, not important variety was seen about the time needed to return to work. But, in Razavi et al. (2003) and Hehenkamp et al. (2006) studies, the return to work and other normal daily routine is lower in the group of UAE [**8**,**16**], which contradicted what we observed.

In this running research, costs related to UAE were larger than these of the myomectomy group. This is in compliance by reports by Pourrat et al. (2003) who reported that UAE costs are larger than trans-vaginal hysterectomy costs [**17**].

According to most studies, a main disadvantage of UAE is the need to re-intervention after five years; Van-Rooij et al. (2005) and Moss et al. (2010) reported the need for re-intervention in a group of UAE compared to the group of hysterectomy and this was mostly in that beginning two-years period after embolization [**11**,**18**]. However, in the running research, not difference was seen among the 2 group regarding the need for re-intervention.

AMH level before and after 6 months post-intervention was studied in the running research. No variety is detected in this regard in either group. When comparing the two groups, also not important variation is detected in the AMH level, as a marker for ovarian capacity. This was in compliance with most studies. In the clinical trial of Hehenkamp et al. (2007), not important variation is detected regarding the FSH level between UAE and hysterectomy groups. After 24 months of follow-up, an important addition in the FSH level is detected in the UAE group. It's to do remarked than women in the mentioned study entered menopause after the intervention [**4**]. Also in the latter study, the AMH level was measured, which was revealed to decrease in all period times, which were anticipated considering the matter that women aged. The research outcomes showed that both UAE and hysterectomy affected the ovarian capacity [**4**]. Rashid et al. (2010) and Mara et al. (2008) measured FSH as a marker for ovarian capacity [**7**,**8**]. The first study reported that among women with FSH levels higher than 40 IU/ L, no difference was seen between UAE and myomectomy, which was compatible with our results [**8**]. In contrast, Mara et al. (2008) who used the cut-off point of 10 IU/ L for FSH, reported that most patients of the UAE group had FSH levels of more than 10 IU/ mL, which is significantly larger while associated to the group of myomectomy [**7**]. Kahn et al. (2011) also showed that the level of FSH is above in the group of UAE associated to myomectomy [**19**], which were not in agreement with our results. 

Gupta, (2012) reported that myomectomy is compared by a better fertility outcome associated to UAE, although this is not important. Not important variation are detected among those 2 methods regarding major complications. Ovarian failure and intervention failure was also comparable between the two methods [**20**]. In Hovsepian et al. (2006) study, even though a continuous rise in the FSH level was seen as a marker for ovarian capacity, no significant difference was seen regarding the FSH between myomectomy, hysterectomy and UAE, groups at 1, 3, and 6 months follow-up [**9**]. In a similar way, no difference was seen regarding the ovarian failure in the 12-months follow-up period among surgery and UAE, at Rashid et al. study (2010) [**8**].

The damage to the ovarian reserve in the two methods of treatment was difficult to diagnose since a single reliable test has not been defined yet. However, regarding the results of the research and similar ones it seems that there is not important variation among UAE and the surgical methods regarding ovarian capacity.

## Conclusion

In general, the outcomes of this research showed that not important variation existed among UAE and myomectomy regarding the hospitalization period, and the time elapsed to return to work which were in compliance by the former studies. Also, it was highlighted that no important variation was seen among the 2 group in terms of the AMH level, what is a marker for ovarian capacity. Therefore, UAE can be utilized to childbearing age females as a less invasive method. A disadvantage of this technique is its high costs.

Considering the low sample size we had, it is suggested that further studies employ more sample size and apply AMH levels, an accurate marker for ovarian capacity. Also, it is better to follow patients for longer periods of time. 

**Acknowledgments**

This article is according to a thesis submitted to the graduate studies office in partial fulfillment of requirements for specialist of gynecology degree and obstetrics by Afsaneh Mansouri in Medical Sciences Kermanshah University, Medicine Faculty.

**Opposition of attention :**

The authors have no opposition of attention to declare
